# Do listeners recover “deleted” final /t/ in German?

**DOI:** 10.3389/fpsyg.2014.00735

**Published:** 2014-07-17

**Authors:** Frank Zimmerer, Henning Reetz

**Affiliations:** ^1^Department of Computational Linguistics and Phonetics, Saarland UniversitySaarbrücken, Germany; ^2^Institute of Empirical Linguistics, Goethe UniversityFrankfurt, Germany

**Keywords:** segment reconstruction, deleted t, perception of deletion, German, natural speech processes

## Abstract

Reduction and deletion processes occur regularly in conversational speech. A segment that is affected by such reduction and deletion processes in many Germanic languages (e.g., Dutch, English, German) is /t/. There are similarities concerning the factors that influence the likelihood of final /t/ to get deleted, such as segmental context. However, speakers of different languages differ with respect to the acoustic cues they leave in the speech signal when they delete final /t/. German speakers usually lengthen a preceding /s/ when they delete final /t/. This article investigates to what extent German listeners are able to reconstruct /t/ when they are presented with fragments of words where final /t/ has been deleted. It aims also at investigating whether the strategies that are used by German depend on the length of /s/, and therefore whether listeners are using language-specific cues. Results of a forced-choice segment detection task suggest that listeners are able to reconstruct deleted final /t/ in about 45% of the times. The length of /s/ plays some role in the reconstruction, however, it does not explain the behavior of German listeners completely.

## Introduction

In normal conversational speech situations, speakers seem to be rather “careless.” One of the most striking results of this careless speech is that speakers often reduce words (e.g., Ernestus, 2000; Johnson, 2004; Zimmerer, 2009). Reductions are rather “minimal” when the number of segments remains unchanged. For instance, segments can be assimilated as in German *Senf* (“mustard”) which may be produced as [zεɱf] instead of [zεnf] (e.g., Zimmerer et al., 2009). Segments can be lenited, for example, medial /t/ may be produced as (reduced) flap in American English (e.g., Kiparsky, 1979; Patterson and Connine, 2001; Connine, 2004; Fukaya and Byrd, 2005; Tucker, 2007; Warner and Tucker, 2011 and references therein). However, reduction processes can alter the pronunciation of words more dramatically. For instance, segments can be deleted, which might even have an impact on the syllabic structure of words (e.g., Johnson, 2004; Zimmerer, 2009).

Generally, it seems that listeners are unimpressed by reductions (including deletions) in normal listening conditions despite the high amount of reductions that speakers produce. It is, however, not yet understood, how they deal with these reductions and deletions, which may be due to the use of ideal, unreduced stimuli (so called “laboratory speech”) in many perception experiments. Although there is a better control over what listeners hear in these circumstances, experiments using exclusively laboratory speech might not tell us how listeners deal with reductions occurring in natural speech. This article aims to better understand the processes that lead to the ease of perception of reduced speech.

A number of studies that used reduced items in perception experiments showed that reduced words seem to be harder to process by listeners, especially if the sentential context is not present (e.g., Pickett and Pollack, 1963; Pollack and Pickett, 1963; Ernestus et al., 2002; Ernestus and Baayen, 2007; Zimmerer, 2009; van de Ven et al., 2011; Zimmerer et al., 2012), which appears to be somewhat contradicting the observation that listeners usually fair well in recognizing what has been said. One possible explanation for the apparent ease of perception of reduced speech is that (at least some of the) segments are reconstructed during the course of perception, due to fine phonetic detail in the input (e.g., Manuel, 1991, 1995; Hawkins, 2003; Niebuhr and Kohler, 2011). This article investigates the reconstruction of a single deleted segment, namely the alveolar voiceless stop /t/ in word-final position in German^1^.

Reconstruction of deleted segments has been shown for several sounds. For instance, Manuel (1991) showed that in words with a deleted Schwa (e.g., a word like *support* sounding like *sport*), fine phonetic cues were used by listeners to differentiate the two words (reduced *support* and *sport*, Manuel, 1991). Similarly, Manuel found evidence that listeners were able to reconstruct deleted /ð/ in nasal contexts, based on fine phonetic detail in synthetically created stimuli. Fine phonetic detail has also been shown to be important for the perception of more massive reductions (e.g., Hawkins, 2003; Niebuhr and Kohler, 2011).

In the literature, language-specific and cross-linguistic tendencies have been identified, both with respect to the production as well as the perception of deleted segments. For instance, the segment /t/ (especially in final position) is very likely to be reduced in Germanic languages (see e.g., Guy, 1980; Neu, 1980; Sumner and Samuel, 2005; Mitterer and Ernestus, 2006; Raymond et al., 2006; Zimmerer, 2009). Concerning this deletion process, researchers have identified many aspects that are very similar across a number of languages as well including aspects of the context, in which /t/ occurs (see next section). However, there are also aspects in production and perception that appear to be language-specific, for instance the role of fine phonetic detail. When speakers leave these cues to signal listeners a deletion in the speech signal, and thereby help them reconstruct segments, they arguably differ across languages, because the phonetic realization of segments (in case of /t/: the amount of aspiration, closure duration, or voicing patterns) and consequently reduction processes are quite different cross-linguistically.

### /t/ deletion in production

The voiceless coronal stop /t/ has been studied intensively. One of the reasons is that the segment is relatively frequent additionally, it is deleted quite regularly in many (Germanic) languages (e.g., Guy, 1980, 1991; Neu, 1980; Mitterer and Ernestus, 2006; Raymond et al., 2006; Zimmerer et al., 2011, 2014)^2^.

Cross-linguistically, (phonological) context proved to be one of the most important factors that can influence the deletion of /t/ (Mitterer and Ernestus, 2006; Raymond et al., 2006; Zimmerer et al., 2011, 2014; Mitterer and Tuinman, 2012). Mitterer and Ernestus (2006), for instance, investigated final /t/ deletion by analyzing two different corpora of spoken Dutch. These corpora differed with respect to both the speech register that was recorded, as well as the vocabulary that had been used. Their results showed that the likelihood of /t/ being deleted in Dutch was highest when preceded by the fricative /s/ and followed by bilabial sounds. Zimmerer et al. (2011), (2014) investigated the deletion of /t/ with help of two newly created corpora that were obtained with a verb paradigm production method which ensured that final /t/ was (at least in one condition) always preceded by /s/, and followed either by /s/, by /v/ or by a vowel. They found overall deletion rates of 20% in the first corpus (Zimmerer et al., 2011) and 27% (Zimmerer et al., 2014) in /-st/ contexts of the second corpus. Concerning context to the right, deletion rates were highest when the /t/ was followed by /s/ and lowest when it was followed by a vowel. Deletion rates were intermediate when final /t/ was followed by the fricative /v/. These results were stable across the two corpora. Similar results were reported by Mitterer and Tuinman (2012) who investigated final /t/ deletion by native Dutch speakers and German speakers of Dutch. The context for final /t/ was either a preceding /n/ or a preceding /s/, and /t/ deletion was most likely when preceded by /s/. However, they found different patterns for verbs and nouns in the two language groups. While Dutch speakers showed the same pattern for verbs and nouns, that is, higher deletion rates after /s/ than after /n/, German speakers behaved (slightly) differently. Deletion rates were higher when the /t/ was preceded by /n/ for verbs produced by German speakers, but with nouns they behaved like Dutch speakers, having more deletions after /s/ than after /n/. Overall, the speakers produced deletion rates of 25% for nouns and 40% for verbs.

Across different studies, other linguistic and extra-linguistic factors have also been identified as influencing the amount of /t/ deletion, such as speaking rate (more deletion in faster speech, cf., Guy, 1980, 1991 see also Byrd and Tan, 1996), speech register (more deletions in less formal speech, cf. Mitterer and Ernestus, 2006), fluency (less deletions in non-fluent speech sections, cf., Raymond et al., 2006; Zimmerer et al., 2011, 2014), social class and dialectal differences (cf., Labov, 1966; Wolfram, 1967), speaker age (tendency for more deletions by younger speakers, cf., Guy, 1991), word category (more deletions in function words than content words, cf., Neu, 1980), relative frequency (the likelihood of /t/ to be deleted was correlated with its likelihood to be decomposed in words like *daftly* and *swiftly*, cf., Hay, 2003). Speaker gender was not consistently found to have an impact on the amount of /t/ deletion [a tendency for more deletions by male speakers compared to female speakers, was found, for instance by Wolfram, 1967; Neu, 1980, but not found by Raymond et al., 2006—analyzing the Buckeye Corpus (Pitt et al., 2007);^3^ or Zimmerer et al., 2014].

The factors that have been found to influence /t/ deletion in different investigations of different Germanic languages seem to point to cross-linguistic similarities, such as segmental context. These similarities concern mainly factors that influence the *amount* or *likelihood* of /t/ deletion. However, the studies reported above also reveal some language-specific differences. These differences mainly concern the *actual realization* of items where the /t/ was deleted. For Dutch, for instance, Mitterer and Ernestus found that speakers kept a preceding /s/ rather short when they deleted /t/. This short /s/ could be interpreted as a cue to a deleted /t/, because in final consonant clusters, such as /st/, the /s/ was produced shorter than if it was a single segment in final position in Dutch (Mitterer and Ernestus, 2006). Interestingly, German speakers used the opposite strategy. When final /t/ in an /-st/ cluster was deleted, German speakers tended to lengthen the preceding /s/ (Zimmerer et al., 2011, 2014). The difference between Dutch and German speakers also raises interesting questions concerning the perception or restoration of deleted /t/ which will be discussed in the next section.

### /t/ deletion in perception

There have been several studies addressing the perception of naturally occurring variants of /t/, including its reduction and deletion. For instance, Sumner and Samuel (2005) investigated released and glottalized variants of final /t/ and their possible role as being represented in long-term memory. When listeners encounter variants of /t/, one could argue that instead of reconstructing the segment, they could also have stored these variants directly. While in their short-term priming experiments all variants activated the correct lexical entry equally well, the results from a long-term priming experiment suggested, that only canonically produced variants (with a full /t/) are stored in long-term memory. These findings indicate that variants with reduced /t/ can be handled well by listeners, but the results not lend support for a direct storage of these variants in long-term memory (Sumner and Samuel, 2005). Furthermore, even if we assume that variants of deleted /t/ were stored in memory, these items could interfere with other words that actually do not have the /t/ in their canonical form. For instance, when the German word *hau-st* (“hit—2nd pers. sg.”) is produced without final /t/, the resulting word will be *Haus* (“house”). This means that listeners would still benefit greatly from strategies to reconstruct deleted /t/ even if (at least some of) the variants were stored in the mental lexicon.

The perception of reduced /t/ was also investigated with its flapped variant. Results were mixed: Some studies showed that flapped variants were perceived (measured as amount of lexical activation) as well as not-flapped variants in American English (Luce and McLennan, 2005; McLennan et al., 2005), other researchers showed that the reduced flap (e.g., unreduced flaps [pʌɾ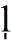] as opposed to reduced [pʌ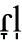]) was not as acceptable in perception than the unreduced flap (e.g., Tucker, 2007). A possible explanation for the difference between these studies is the status that has been assigned to the flap. Tucker assumed the flap version as canonical and only the reduced flap version as a reduced variant, McLennan and colleagues treated the flap as a reduced variant of an underlying /t/.

Concerning deleted /t/, Mitterer and Ernestus (2006) investigated the extent to which the (acoustic) patterns that were produced by speakers of Dutch—the relatively short /s/ as a cue to an underlying final consonant cluster (see /t/ deletion in production)—had an impact on perception of deleted /t/. They investigated in a perception study with resynthesized stimuli whether Dutch listeners were able to use the cues of a short /s/ to reconstruct final /t/. Furthermore, Mitterer and Ernestus were interested whether there is a difference in /t/ reconstruction depending on the context, that is, whether in the /s/ context, where /t/ deletion occurs more often, Dutch listeners are more likely to reconstruct /t/ than in the /n/ context, which is not as prone to the deletion of /t/. The findings of Mitterer and Ernestus suggest that this is indeed the case. Dutch listeners are more likely to reconstruct /t/ in the /s/ context than in the /n/ context (see also Mitterer and McQueen, 2009 for similar results), and the listeners seem to use the cue of /s/ shortness to reconstruct final /t/. This also suggests that listeners are well aware of production patterns and use this information in the perception of deleted /t/.

In another study, Mitterer and Tuinman (2012) investigated the extent to which the cues left in the acoustic signal are language-specific for the reconstruction of final /t/ with native Dutch participants and German learners of Dutch. For stimuli similar to the ones used in Mitterer and Ernestus (2006), listeners were most likely to perceive a word containing /t/ the more evidence for /t/ was present. Also, participants perceived more often a /t/, if a possible reconstruction created a word, and if the preceding context was /s/. However, there were also differences between German and Dutch listeners. German listeners were in some cases overgeneralizing in the reconstruction of /t/. Mitterer and Tuinman interpreted the difference in reconstruction rate as partly being conditioned by transfer from their German native language, where /t/ deletion is overall less frequent, and where the cues for deleted /t/ may be different. This raises the question of the use of language-specific cues for reconstruction of /t/, which will be addressed in this article.

### Research questions

The short overview over production and perception of deleted /t/ suggests that there are both cross-linguistic tendencies, such as context of final /t/, and language-specific processes. This article aims to investigate to what extent perception and reconstruction of /t/ deletion in German is language specific. Two research questions are addressed with a forced-choice segment detection method:

- To what extent are German listeners able to reconstruct seemingly deleted /t/? In German, about 20% of final /t/s get deleted (e.g., Zimmerer, 2009; Zimmerer et al., 2011, 2014). This means that listeners are faced with the process of /t/ deletion quite frequently.
- The second question addresses the language-specific aspect of reconstruction. Dutch listeners have been shown to use the fine phonetic details, such as /s/ length as cues for reconstruction (e.g., Mitterer and Ernestus, 2006; Mitterer et al., 2008). The findings of Zimmerer and colleagues suggest that the length cue is different in German compared to Dutch, and the results of Mitterer and Tuinman (2012) further indicate that perception might be different for German and Dutch listeners. Therefore, the question arises whether German listeners are aware of the /s/ length cue and use it to reconstruct deleted /t/.

## Materials and methods

### Participants

In all, 14 native (11 female) German speakers participated in the experiment. They were between 20 and 34 years old (mean 24.9) and were recruited at the Goethe-University, Frankfurt. They received payment in kind (cookies and coffee/tee/juice) for their participation. All of the participants spoke standard German and were born in Hesse, or Northrhine-Westphalia. All of them had lived for more than 5 years in Frankfurt area. None of the participants reported any hearing problems. They were naïve with respect to the purpose of the study, and were told about the process of /t/ reconstruction only after they completed the experiment. An experimental session lasted about 25 min, including reading the instructions and the practice session.

### Materials

The basis for the experimental stimuli were utterances recorded for a verb paradigm production corpus (Zimmerer et al., 2014). The corpus consists of paradigm productions of 50 different German verb forms. Participants had produced paradigmatic cells of verbs, where a verb form was always preceded by its correct pronoun. For instance, for the verb *hauen* (“hit-inf”), a paradigmatic cell was “*du haust, sie haut, ihr haut, sie hauen*” (“you-2nd pers sg. hit, she hits, you-2nd pers. pl. hit, they hit”)—the context where /t/ deletion can occur is the second person singular ending of the person/number suffix—st (e.g., *du hau-st* “you 2nd pers. sg. hit”), which is underlined. In some cases, 3rd person singular renditions can also end in /st/, however, the morphological structure of these forms is different (e.g., *er haus-t* “he dwells”).

All items used in the forced-choice experiment were either fragments of verbs or fragments of verbs that were followed by (fragments of) pronouns from this corpus. All items were excised with Praat (Boersma and Weenink, 2013). For deleted /t/ items, fragments were extracted from the paradigms that included the vowel of the verb stem, followed by /s/, and the subsequent word could begin in either [si] (from *sie* “she/they”) or /vi/ (from *wir*—“we”)^4^. This procedure ensured that the items did not sound word-like. Therefore, possible /t/-decisions for /t/-deleted items (henceforth *Øt-items*) were not based on lexical influence, where listeners could have reconstructed a /t/ more often in order to create an existing word form in German (e.g., Ganong, 1980; Mitterer and Tuinman, 2012).

In a pretest, 93 *Øt-items* from the corpus were presented to 5 listeners for transliteration, all were students of the institute of Phonetics in Frankfurt. They were asked to write down what they heard, and had 4 s to give a transcription for every item, which they could listen to only once. The instructions were kept very simple (i.e., “write down what you think you heard”), without mentioning the segment /t/ or any reduction process or the fact that they were excised from verb paradigm productions. For the experiment reported here, 30 items which were transliterated without /t/ were used (i.e., an item counted as being transliterated by all listeners without /t/ when it was written with neither “t” nor “z”)^5^. This procedure ensured that items were used which were rated /t/-less when no close attention was paid to the segment /t/.

For the experiment, a total of 180 stimuli were excised from this verb paradigm production corpus (Table 1 gives an overview over the segmental make-up of the stimuli). Overall, 90 of these items had a /t/ present (+*t-items*). These were 45 stimuli with a /t/ (*t-items*) intervocalically, such as [aːtə], a fragment based on *braten* (“fry-inf”), and further 45 stimuli which had /t/ occurring in an /s/ context, that is, a /ts/ (which also counted as a /t/) like in [iːstsi] from “… *fliehst, sie* …” (“flee 2nd pers. sg., she”) (*ts-items*). Note that the affricate /ts/ is a phoneme of German which is written “z” in orthography (see also footnote 5). The segment sequence [ts] in the *ts-items* could possibly also interpreted as an affricate, which could have repercussions for the results (see section Results and Discussion and Conclusions). However, these items were included in order to have [s] present in all experimental conditions and not only in the −t and Øt conditions. Furthermore, the inclusion of these items is very close to the segmental and syllabic structure of the *Øt-items*. Then there were 60 items without an underlying /t/ (*−t-items*). These were 30 stimuli which had a fricative /s/ or /z/ preceded and followed by a vowel (*s-items*) such as [iːsə] excised from *flieβen* (“flow-inf”) and 30 stimuli which had another consonant (e.g., /n/), intervocalically (*n-items*) such as [anə] which was part of *bannen* (“ban-inf”). Finally, the third group (30 stimuli) had the /t/ deleted (*Øt-items*). One example is a fragment from the paradigm where in “*… fliehst, er* … ” (“flee 2nd pers. sg., he”) the /t/ was deleted and the fragment was [iːse]. By definition, in the *Øt-items*, /t/ was deleted word finally and followed by either a consonant (/v/ or /s/), or by a vowel if the deletion lead to a sequence of two /ss/ where no boundaries between the segments could be established which were treated as one single /s/. As can be seen in Table 1, the number of *+t-items* is higher than the number of *−t-items*. Because the *Øt-items* were transcribed without “t” in the pretest, and we did not know to what extent German listeners would be reconstructing “t,” we counted these as instances of t-less items as well. This led to 90 items with “t” and 90 items without “t.”

**Table 1:** **Stimuli in the experimental conditions**.

**Experimental condition**	**Item-type**	**CVC–structure**	**Example in IPA**	**Mean duration**	**No. of stimuli**
+t	t-items	VtV	[aɱte]	262 ms	45
	ts-items	V(C)tsV	[iɱstsi]	423 ms	45
−t	s-items	VsV	[iɱsə]	320 ms	30
	n-items	VCV	[ane]	256 ms	30
Øt	Øt-items	Vst-(C)V	[iɱse]	392 ms	30
SUM				332 ms	180

The 180 items used for the experiment were produced by ten speakers of the verb paradigm production corpus mentioned above (Zimmerer et al., 2014). Individual speakers contributed between 11 and 22 items for the experiment. These speakers were also students at the Goethe-University who spoke standard German and had spent at least 5 years in Frankfurt. None of the speakers participated in the perception experiment.

### Design and procedure

An experimental trial consisted of a warning tone, which was followed by 250 ms of silence. Then, the items were presented, after which participants had 1500 ms to decide whether the item they heard had a /t/ present or not, before a new trial began. Participants were asked to press the respective response button (“t” or “no t”) on a response box with their dominant index finger. Response measurements were conducted with help of a custom-made software and hardware combination (Reetz and Kleinmann, 2003) where response boxes (with two responses buttons labeled “t” and “no t”) were connected to an external device and subsequently saved onto an Apple Mac Book Pro. Participants were tested wearing Sennheiser eH-350 headphones.

Three experimental lists were created with the 180 items. Each list was pseudo-randomized and between the experimental lists, participants could take a break. Overall, they decided 540 times whether a fragment had a /t/ present or not. Participants were tested in groups of four or less. They received written instructions before the experiments. They were asked to respond as quickly and as accurately as possible, whether the fragment they heard had a “t-sound” present or not. Participants received a training section with 14 items that were not part of the experiment. The training section preceded the actual experiment to familiarize the participants with the task and the procedure of the experiment.

Accuracy rates were calculated as percent correct responses for the respective category (after exclusion of no responses and responses that were too fast or too slow).

### Predictions

- If the /t/ was indeed completely deleted (*Øt-items*), participants should treat these items as they would treat items where no /t/ was present (*−t-items*). However, based on the results reported by Mitterer and colleagues (Mitterer and Ernestus, 2006; Mitterer and Tuinman, 2012) and based on the reconstruction of other segments, listeners may be able to use the cues that are left in the speech signal to reconstruct /t/ in some cases, and we expect some qualitative difference compared to items where no /t/ is present (*−t-items*). We do not expect reconstruction rates that are identical to underlying items (*–t- or* +*t-items*).
- For the items where no /t/ was present or where there was an underlying /t/ (i.e., −*items and +t-items*), we expect very high accuracy rates, because the task itself is rather straightforward and it should not lead to high error rates.

## Results

In a first analysis, we investigated how accurate participants were responding to the fragments they had to listen to. Each of the participants was expected to respond to 540 fragments. Therefore, 7560 responses were expected (3 repetitions × 180 items × 14 listeners). Before examining the reconstruction of /t/, it is important to see how participants responded to clear cases where a /t/ was present or where no /t/ was present (i.e., the *+t- and −t-items*). The responses to +*t-items* and *−t-items* were also used to potentially exclude participants that showed a high error rate in these response categories. For the two underlying categories, 6300 responses were expected (3 repetitions × (90 + 60) items × 14 listeners). Of these, there were 64 cases where no response was given which were excluded from further analysis. Furthermore, 117 cases of responses that were faster than 150 ms and slower than 900 ms were also excluded. Wrong responses occurred when participants pressed “t” for *−t-items* or “no t” for +*t-items* (217 responses—20 *s-items*, 19 *n-items*, 22 *t-items*, 156 *ts-items*). For the statistical analysis, errors were coded with “0” and correct responses with “1.” Table 2 gives an overview over the accuracy rates [Least Square Means (LSM) as well as Means] for the *+t-* and *−t*-*items*. Accuracy rates for individual participants ranged between 99.3 and 90.7%; no participant was excluded. For the analysis, a linear mixed model was calculated with JMP (SAS, 2012), with item and participant as random factors, item-condition (*t-item, ts-item, s-item, n-item*) as factor, and accuracy as dependent variable. Results indicate that item-conditions differed significantly [*F*_(3, 144.2)_ = 30.47; *p* < 0.0001]. This was driven by the lower accuracy rate for *ts-items* that were significantly different from all other item-conditions, which did not differ significantly, as was indicated by a Tukey HSD *post-hoc* test.

**Table 2:** **Least Square Means (LSM) and Means of Accuracy for the underlying items^a^**.

**Experimental condition**	**Item-type**	**LSM accuracy**	***SE***	**Mean**	***SE***
+t	t-items	0.988	0.009	0.988	0.004
	ts-items	0.913	0.009	0.913	0.004
−t	s-items	0.983	0.01	0.984	0.005
	n-items	0.985	0.01	0.985	0.005

a *Accuracy was calculated as percentage of correct responses to the items of the respective category*.

For the next analysis, that is, the comparison with *Øt-items*, there was no difference made between *t-items* and *ts-items* (i.e., the *+t-items*) on the one hand and *s-items* and *n-items* (i.e., the *−t-items*) on the other hand.

**Figure 1:**
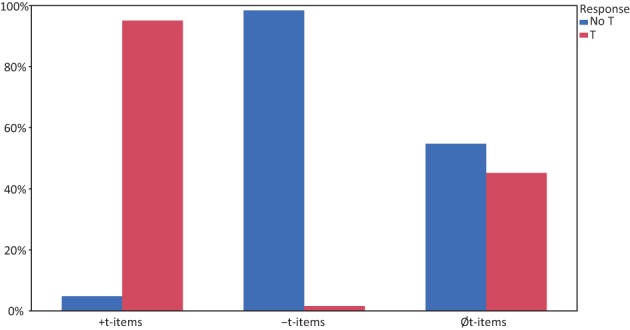
**Percent of different responses to the respective categories, “no t” responses are in blue, while “t” responses are depicted in red**.

In a next step, response categories for *Øt-items* were compared to responses to the two underlying item categories. For this analysis, cases where no response was given (91 responses) or responses that were too fast or too slow (154 responses) were excluded from further analysis. *Øt-items* were responded to with “t” in 45% of the time (541 responses), whereas they received a “no t” response in 655 cases (55%). For statistical analysis, we treated “no t” responses to *Øt-items* as “correct” to be able to compare the results with the underlying items in a linear mixed model. Figure 1 shows the responses given to the respective categories (*+t-, −t*- and *Øt-items*), whereas Table 3 reports the LSM for the respective categories. The linear mixed model with item and participant as random factors, item-condition (*+t*, −*t, Øt*) as factor and accuracy (“0” for incorrect, “1” for correct) as dependent variable showed that item-condition was a significant factor [*F*_(2, 176.4)_ = 223.84; *p* < 0.0001], and that each of the three conditions differed significantly. This means that deleted /t/ was reconstructed in a little less than half of the possible cases.

**Table 3:** **Least Square Means (LSM) of Accuracy for different item categories**.

**Experimental condition**	**LSM accuracy**	***SE***
+t	0.95	0.013
−t	0.98	0.015
Øt	0.55	0.02

As indicated in the Materials and Method section, the number of stimuli was not evenly distributed for the three conditions (i.e., 90 *+t-items*, 60 *−t-items* and 30 *Øt-items*). This could have led participants to create a response bias. Therefore, we also used d' as a way to account for possible response biases (e.g., Stanislaw and Todorov, 1999; Macmillan and Creelman, 2005)^6^. The d' values were analyzed to compare sensitivity differences for the different items. A linear mixed model was calculated with participant as random factor and comparison as fixed factor, as well as d, as dependent variable. Results indicate that comparison is a significant factor [*F*_(2, 26)_ = 74.9; *p* < 0.0001]. A Tukey HSD test showed that all comparisons were different from each other. Table 4 indicates the LMS for the different comparisons in this analysis. The d' analysis thus shows the same tendency as the accuracy ratio analysis.

**Table 4:** **Least Square Means (LSM) of d' in the respective comparisons**.

**Experimental condition**	**LSM accuracy (d')**	***SE***
−t vs. +t	3.98	0.17
−t vs. Øt	2.39	0.17
Øt vs. +t	1.87	0.17

**Figure 2:**
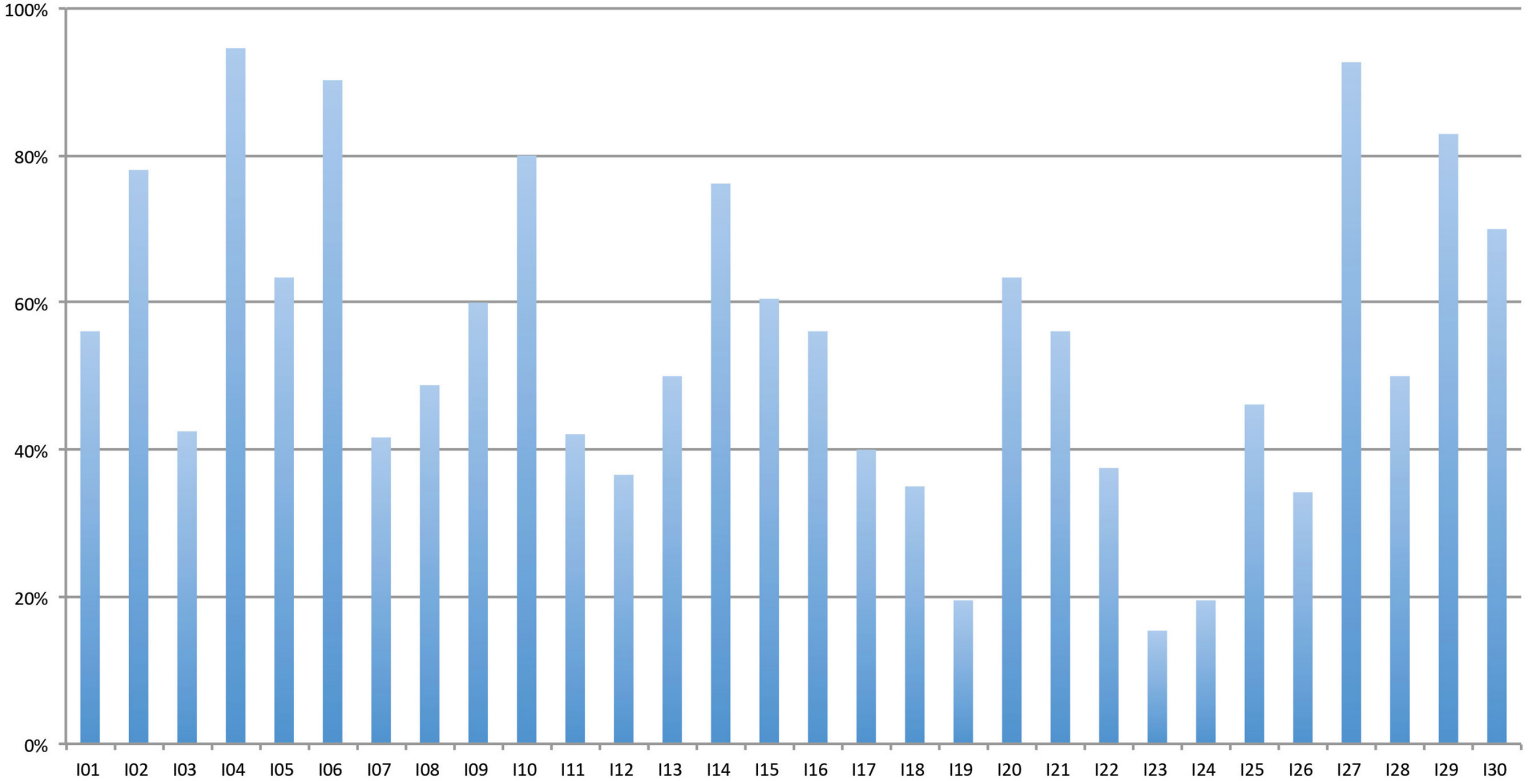
**Percent “no t” responses to the different *Øt-items***.

A closer inspection of the different items showed that there was considerable variation between individual *Øt-items*. The amount of “no t” responses ranged from 95 to 15.4%. Figure 2 shows the percent “no t” responses for each of the *Øt-items*.

**Figure 3:**
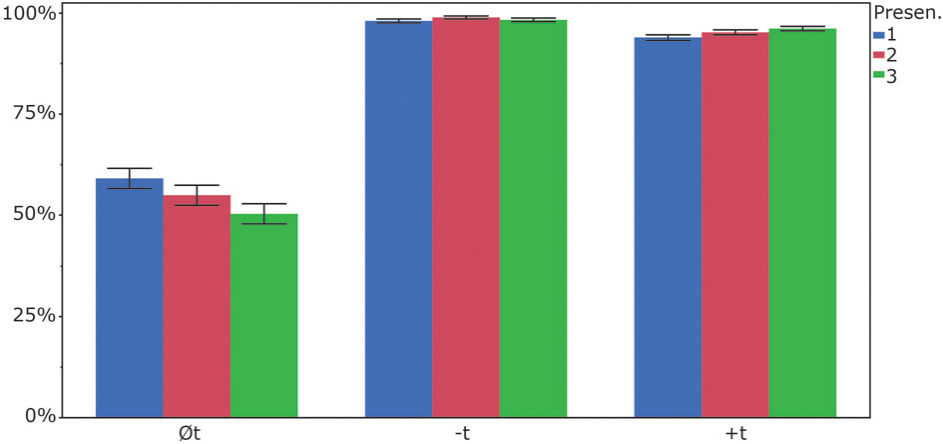
**Percent correct responses (“no t” for *Øt* and −*t-items*; “t” for +*t-items*) for the three presentations**.

In a next step, we analyzed whether participants showed different responses to the items in the course of the experiment, that is, whether they changed their reconstruction behavior over time. To this end, we calculated a linear mixed model with item and participant as random factors, item-condition (*+t, −t, Øt*), presentation (*first, second, third*) and the interaction of item-condition and presentation as fixed factors and accuracy (“0” for incorrect, “1” for correct) as dependent variable. The results show that item-condition was a significant factor [*F*_(2, 176.5)_ = 22.94, *p* < 0.0001], as was presentation [*F*_(2, 7118)_ = 3.82, *p* < 0.05]. The interaction item-condition × presentation turned out to be significant as well [*F*_(4, 7117)_ = 8.07, *p* < 0.0001]. In this model, concerning item-condition, *Øt-items* differed from the other two item groups, but +*t-items* and *−t*-*items* items did not differ significantly from each other. When we look at the factor presentation, the first presentation had an overall accuracy rate of 83.6%, the second presentation was responded to correctly in 83% of the cases, whereas in the third presentation, participants responded correctly in 81.5% of the cases (See Figure 3). A Tukey HSD *post-hoc* test showed that the third condition was significantly different from the first one, but the first presentation was not different from the second, nor was the second from the third. Finally, the significant interaction of item-condition × presentation was driven by the fact that the *Øt-items* showed different accuracy rates in the three presentations. Participants responded with “no t” to *Øt-items* in 59.1% during the first presentation, 54.9% during the second presentation and 50.2% during the third one. The Tukey HSD *post-hoc* analysis indicates that the first and third presentations of *Øt-items* were different from each other, but the first and second were not, nor were the second and the third. The *+t-items* and the *−t-items* did not show significant differences in the three presentations, but were significantly different from all *Øt-item* presentations.

A final investigation analyzed a possible correlation between the reconstruction of /t/ and the s-length of the *Øt-items*. The correlation that was found was significant but not very strong [*r*^2^_(2)_ = 0.03; *p* < 0.0001]. The analysis showed that the longer the /s/ in the signal, the more likely were participants to reconstruct /t/.

## Discussion and conclusions

The first research question we investigated in this article concerns the extent to which German listeners are able to reconstruct seemingly deleted /t/. In German, about 20% of final /t/s get deleted (e.g., Zimmerer, 2009; Zimmerer et al., 2011, 2014). If German listeners behaved similar to Dutch listeners, reconstruction should be frequent, but it should also not occur in every instance.

The results from a forced choice phoneme detection task indicate that listeners are at least sometimes able to do reconstruct deleted /t/. When they were faced with fragments from speech with a deleted /t/, reconstruction occurred in about 45% of the cases. The amount of /t/ responses for the *Øt-items* is clearly different from both *+t-items* (with more than 95% “t” responses), as well as from *−t-items* (with less than 5% “t” responses). This finding is also supported by the analysis of d, where *Øt-items* fell in between the clear cases of *−t-* and *+t-items*.

Thus, compared to Dutch listeners, Germans seem to reconstruct deleted /t/ less often (cf. Mitterer and Ernestus, 2006; Mitterer and Tuinman, 2012). One explanation for this difference is the choice of stimuli in the experiment reported here, which are different from the experiments reported by Mitterer and colleagues. Mitterer and Ernestus used synthetically manipulated stimuli in sentential contexts (as did Mitterer and Tuinman), whereas in this study, only fragments from verbs and pronouns, that is, parts of real speech were used. These fragments were presented without context and were not word-like. Therefore, the stimuli in this study were on the one hand arguably closer to the kind of speech that is encountered by listeners in natural situations, since no additional manipulation (e.g., synthesis) was performed. On the other hand, they were also less natural, since usually, speech never occurs without context. Furthermore, the stimuli used here allowed for less control over what cues speakers could have left for deleted /t/ (see below). A third explanation for the different results could be that the stimuli here were not word-like, and thus, prevented listeners to adjust their perception by reconstructing a /t/. German listeners were shown to rely more on higher-level lexical knowledge when reconstructing /t/ in their foreign language Dutch (Mitterer and Tuinman, 2012). Also, in “normal” speech, German verbs are produced with a pronoun. That pronoun additionally helps to perceive the correct word and to activate the intended meaning. Therefore, this study can be seen to indirectly show further evidence for the importance of context when listeners have to deal with deletions. If context is missing, listeners have been shown to need additional effort for successful recognition (e.g., Pickett and Pollack, 1963; Pollack and Pickett, 1963; Ernestus and Baayen, 2007; Zimmerer, 2009; van de Ven et al., 2011; Zimmerer et al., 2012). To some extent, the lower reconstruction rates of German listeners could be partly due to language-specific behavior, too. Production analyses revealed a slightly higher amount of /t/ deletion in Dutch compared to German. Therefore, Dutch listeners are faced with the deletion of /t/ more often and therefore might have a reconstruction strategy that is based more on phonetic cues (such as /s/ length or sentential context), whereas German listeners are more focused on the lexical information for reconstructing /t/.

This interpretation is also connected to the second research question we addressed in this article, concerning the impact of length of /s/ preceding a deleted /t/ on the reconstruction of /t/ (which seems to be language-specific). Dutch listeners have been shown to use the fine phonetic details of /s/ length as cues for reconstruction (e.g., Mitterer and Ernestus, 2006; Mitterer et al., 2008), but results from production analyses of German speakers indicate that German speakers behave differently from Dutch speakers, therefore, reconstruction strategies might also be different for German and Dutch listeners. The results of this study suggest that despite the consistent production of /s/ lengthening by German speakers, there seems to be only a small, but significant correlation between /s/ length and /t/ reconstruction. On the one hand, this finding can be seen as another argument in favor of language-specific reconstruction strategies of German listeners when encountering deleted final /t/. At the same time, the rather small correlation indicates that listeners are not focusing solely on /s/ length when they reconstruct /t/. The rather small effect of /s/ length could be explained partly by the nature of the stimuli (i.e., fragments without or with only minimal context). Another possible explanation is the number of stimuli with deleted /t/, which is quite small. The stimuli were all excised from the corpus without any consideration to /s/ length. Despite the consistent lengthening of /s/ in case of /t/ deletion, there is also overlap between the length of /s/ in cases where /t/ is deleted and where /t/ is not deleted (cf. Zimmerer et al., 2011, 2014). Therefore, listeners cannot be completely sure about the nature of the /s/ in the stimuli they encounter in the experiment. Furthermore, the fragments were rather short and speech rate (which may play an important role for the perception of /s/ length) cannot be estimated with high confidence by listeners, which might prevent them from using the cue of /s/ length consistently.

One result of the experiment that could lead to further research in the future concerns the differences for the underlying categories with respect to accuracy. The *ts-items* were more difficult for the participants to respond to correctly than the *t-items*, but apart from these, there was no difference between the other underlying categories. A possible explanation is that /t/ may be acoustically less salient when preceding /s/. This point is actually one of the explanations why /t/ gets deleted in such contexts in the first place. The *ts-items* were similar to *Øt-items* concerning the segmental structure, and if deletion is regarded as extreme form of reduction, maybe some of the *ts-items* were reduced to some extent. However, we also cannot rule out an orthographic influence, because in German orthography, [ts] can be written in some cases as “ts” or as “z” in others. Therefore, a transfer to the decision “t” or “no t” may be additionally difficult in these cases. Listeners could have treated these items as underlying affricates and thus refrained from responding with “t.” Especially this last possibility should be investigated in future research. It would be interesting to find out, whether listeners treat sequences of the segments [t] and [s] which are parts of the fragments across word boundaries the same or differently from underlying affricates [ts], in words like *Mütze* (“cap”) or *Herz* (“heart”), which could also be tested for the influence of orthography, because in some cases the “t” is written, in others not.

An effect that also emerged in the results was that participants responded to *Øt-items* more often with “t” in the third presentation compared to the first one. Possibly, this shows that listeners were able to learn during the experiment and focus on phonetic cues that were left in the speech signal. Despite the overall tendency to reconstruct deleted /t/ more often later in the experiment, not all items showed this trend. Individual items showed very different patterns across the three conditions some had also more “t” responses in the first presentation compared to the last one. The very different patterns of reconstruction and the finding that, overall, more deleted /t/s were reconstructed during the third presentation compared to the first one may also indicate that the overall asymmetry was not a decisive factor. If this was the case, we would have found more likely the reverse pattern. Because the number of *+t-items* is higher than *−t-items*, participants may have a “no t” bias in their response to press each button equally often. If listeners had the expectation that they should give an equal number of “t” and “no t” responses, we would expect even more “no t” responses during the third presentation, because the asymmetry would have built up an increased response bias. This was not the case, however. Taken all presentations together, /t/ was not reconstructed in the majority of the cases by the participants.

The fact that listeners are not able to restore /t/ consistently is also shown by the varying success in /t/ restoration for individual stimuli, showing a range between 15 and 95%. No item was always (or never) restored and the variation between the items is considerable. At this point, we can only speculate about the cues that were left in the items which were leading to a more successful reconstruction compared to other cues, because /s/ length does not seem to be capturing the whole picture. Despite the significant correlation, it is extremely week. This finding also leads to a possible extension of the experiment for further research. The choice of stimuli in this experiment was based on a pretest, where only stimuli were chosen which were never responded to with a /t/ sound. However, since reduction has been shown to be gradient, it could be interesting to include also items which were transcribed with /t/ in some of the cases in the pretest. Arguably, in these items, more cues for /t/ were left in the speech signal. The inclusion of such items could shed more light on the question what exactly the cues are and could also be used to investigate gradient activation (cf. Janse et al., 2007).

A question that additionally could arise with respect to the pretest is the difference concerning the amount of reconstruction in the pretest and in the phoneme detection experiment. At this point, we can offer only speculative explanations. First, the number of participants in the pretest was very small. Therefore, it could be possible that with more transcribers, more “t” transcriptions could have occurred for the items we chose as *Ø-items*. Furthermore, the participants for the pretest listened to each stimulus only once, and they were completely unaware of the purpose of their transcriptions and therefore did not pay attention to /t/. In the forced-choice experiment, however, participants concentrated on the presence of /t/. They had to make the decision, whether due to some phonetic detail, there could have been a /t/ present in the stimulus, and they had to do so under some time-pressure. In some cases, they arguably made a “random” decision (nearly half of the 30 *Øt-items* had accuracy rates in the range of 40–60%, cf. Figure 2). This attention to fine phonetic detail to come to a decision may also result in an overall increase in the likelihood to reconstruct /t/. And because participants were told that there is either a “t” sound present (or not), they had to decide spontaneously; there was no third option. Actually, in this respect, the rather unnatural task (“press a button for “no t” or “t”) might be even somewhat close to natural speech situation, because in these, especially, when possible ambiguities are created by the deletion of segments, listeners also have to decide (under time pressure, because conversation goes on), whether there was a /t/ present or not, and they might use all the cues they can to come to that decision (including sentential context, of course, which was absent here). At this point, we cannot be sure, which of the explanations is most accurate and some of the arguments might seem speculative, but this question is also an interesting methodological field for further research. Mitterer and colleagues circumvented many of possible problems with the stimuli by using resynthesized stimuli where it is possible to control very tightly the acoustics of what is presented. The use of non-manipulated stimuli in our experiment does not allow for such a control. At the same time, non-manipulated stimuli might be closer to what listeners are faced with in natural occurring speech. For future research, it may be important to use both natural stimuli excised from corpora with stimuli that were artificially resynthesized.

### Conflict of interest statement

The authors declare that the research was conducted in the absence of any commercial or financial relationships that could be construed as a potential conflict of interest.

## Footnotes

^1^Note, that we call the process (/t/) deletion, despite some evidence that listeners produce some phonetic cues that could be interpreted as remnants of /t/. For us, items that were transcribed as /t/-less in the corpora we used in this investigation are called deleted. This is done to differentiate this process from reductions like flapping that is more regular. Furthermore, we apply this strategy also to other cases from the literature (e.g., Manuel, 1991) which are sometimes called “seemingly” deleted.

^2^Regularity in this case refers to the fact that the deletions occur in noteworthy percentage of the possible cases. This does not necessarily indicate a phonological regularity. Note, however, that the notion of variable rules has been used to describe the process (e.g., Guy, 1980; see also Cedergen and Sankoff, 1974).

^3^They also investigated deletion of medial /d/.

^4^In a canonical standard German production, the pronoun sie (“she”) should be produced as [zi:]. However, in conversational speech, the initial /z/ is very often devoiced (cf. the Kiel Corpus of spontaneous speech, IPDS, 1994). Only [s] was produced in the items that were used for the experiment.

^5^In German orthography the letter “z” denotes the affricate [ts].

^6^In cases where participants had an accuracy rate of 100% (this occurred for −*t-items* only, we set the hit rate to 0.99 by adding one miss to the performance—this occurred for 7 of the participants).
